# Diagnostic yield of NanoString nCounter FusionPlex profiling in soft tissue tumors

**DOI:** 10.1002/gcc.22834

**Published:** 2020-01-31

**Authors:** Wangzhao Song, Inge Platteel, Albert J. H. Suurmeijer, Léon C. van Kempen

**Affiliations:** ^1^ Department of Pathology and Medical Biology University Medical Center Groningen, University of Groningen Groningen The Netherlands

**Keywords:** fusion genes, molecular pathology, NanoString, sarcoma, soft tissue tumor

## Abstract

Diagnostic histopathology of soft tissue tumors can be troublesome as many entities are quite rare and have overlapping morphologic features. Many soft tissue tumors harbor tumor‐defining gene translocations, which may provide an important ancillary tool for tumor diagnosis. The NanoString nCounter platform enables multiplex detection of pre‐defined gene fusion transcripts in formalin‐fixed and paraffin‐embedded tissue. A cohort of 104 soft tissue tumors representing 20 different histological types was analyzed for the expression of 174 unique gene fusion transcripts. A tumor‐defining gene fusion transcript was detected in 60 cases (58%). Sensitivity and specificity of the NanoString assay calculated against the result of an alternative molecular method were 85% and 100%, respectively. Highest diagnostic coverage was obtained for Ewing sarcoma, synovial sarcoma, myxoid liposarcoma, alveolar rhabdomyosarcoma, and desmoplastic small round cell tumor. For these tumor types, the NanoString assay is a rapid, cost‐effective, sensitive, and specific ancillary screening tool for molecular diagnosis. For other sarcomas, additional molecular testing may be required when a translocation transcript is not identified with the current 174 gene fusion panel.

## 1 INTRODUCTION

Soft tissue tumors represent a remarkably heterogeneous group of neoplasms, with many subtypes being exceptionally rare. More than 100 different soft tissue tumors have been described in the latest 2013 WHO classification.^1^ The proper histological classification of soft tissue tumors is grounded in the microscopic analysis of tumor growth patterns and their cytological features, which may be a difficult exercise, since many tumors have overlapping morphologic features. Although tumor‐associated protein markers may be visualized by ancillary immunohistochemistry (IHC), many tumors show nonspecific, overlapping or absent marker expression. Thus, it may be difficult or impossible to render an objective accurate diagnosis, in particular when studying small biopsy specimens with a limited amount of tumor tissue.

Fortunately, a significant number of soft tissue tumors, in particular those with monomorphic round cell, spindle cell or epithelioid morphology, harbor recurrent gene translocations, which are often tumor‐specific. These unique recurrent translocations were first discovered in the early 1990s by chromosomal banding techniques, for example, the *t(X;18)(p11;q11)* translocation in synovial sarcoma, which results in the tumor specific *SS18‐SSX* fusion genes.^2^ At the molecular level, with knowledge of the exon regions involved in fusion genes, RT‐PCR and Fluorescence In Situ Hybridisation (FISH, using break‐apart probes) methods became available to detect these particular gene fusions and rearrangements. In the past decade, pathologists have witnessed the rapid development of next generation sequencing (NGS) techniques, which allow simultaneous detection of multiple fusion transcripts. This translated into more accurate classification and also prognostication of soft tissue tumors.^3^ At present, the two novel molecular multiplex methods commonly used in Dutch sarcoma centers are the anchored multiplex PCR (AMP)‐based NGS (Archer FusionPlex Sarcoma assay)^4^ and the NanoString nCounter platform.^5^ The Archer AMP PCR method targets exons of 26 genes commonly involved in fusion genes of soft tissue tumors, whereas the NanoString assay is a high‐throughput hybridization technique, which uses specific probes that target 174 unique gene fusion junctions in 22 soft tissue tumor types.^6^

In this quality control study, we evaluated the sensitivity and specificity of the NanoString nCounter platform for gene fusion detection in 22 different soft tissue tumors, adding our results to the initial report on this method.^7^

## 2 MATERIALS AND METHODS

### 2.1 Case selection

**Figure 1:**
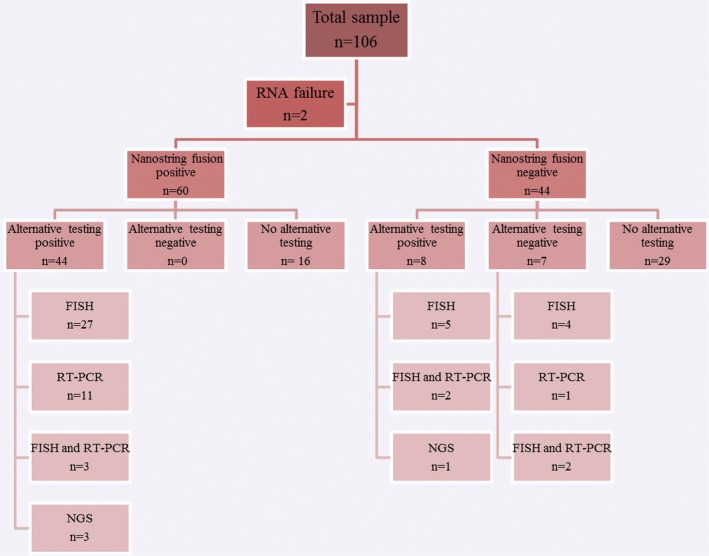
Overview of Nanostring nCounter FusionPlex results [Color figure can be viewed at http://wileyonlinelibrary.com]

Case selection included 106 soft tissue tumors derived from the archives of the Department of Pathology in the University Medical Center Groningen and diagnosed between 1988 and 2018. The series comprised 22 different translocation‐associated tumor types. All cases were reviewed by a pathologist with special expertise in diagnostic pathology of soft tissue tumors (A.S.). In all cases, Formalin‐Fixed and Paraffin‐embedded (FFPE) material was available, in the large majority of cases from tumor excision or resection specimens. In two tumor specimens (one undifferentiated round cell sarcoma and one desmoplastic small round cell tumor), RNA quantity was too low to allow proper analysis. Thus, 104 tumors were eventually included in the study, of which 59 tumors had been tested previously by an alternative molecular method (Figure 1), including FISH (36 cases), RT‐PCR (12 cases), FISH and RT‐PCR (7 cases), or Archer NGS (4 cases). Fifty‐two out of fifty‐nine cases were fusion positive by alternative molecular tests. In the remaining 45 cases, in which no molecular methods had been applied, the tumor diagnosis was based on clinical presentation and histologic features in combination with IHC.

The study was approved by the UMCG institutional ethical review board (P18‐116) and performed in accordance with the code of conduct for responsible use of human tissue that is used in the Netherlands (Dutch Federation of Biomedical Scientific Societies; http://www.federa.org).

### 2.2 NanoString gene expression profiling

RNA was isolated from four 5‐μm‐thick formalin‐fixed and paraffin‐embedded tissue sections containing at least 50% tumor cells using the RNeasy mini kit (Qiagen) according to suppliers instructions. Total RNA was quantified with Qubit (ThermoFisher).

The soft tissue and bone tumor probe set as described by Chang et al.^7^ was ordered from IDT Technologies (Leuven, Belgium). In contrast to the initial study, our panel did not contain probes for the detection of *COL1A1*‐*PDGFB* gene fusion transcripts, as can be found in dermatofibrosarcoma protuberans. Probes were hybridized with 100 ng RNA overnight in a thermocycler at 67°C with a heated‐lid at 72°C. The RNA‐probe complexes were loaded on an nCounter cartridge, and hybridized, washed and read on a nCounter SPRINT platform according to suppliers instructions (NanoString nCounter Technologies, Seattle, WA).

### 2.3 Data analysis

The platform‐generated Reporter Code Count (RCC) files containing the raw data were analyzed. Samples with a geometric mean of the raw counts of the four reference genes (*ACTB*, *GAPDH*, *SDHA*, *UBC*) of <500 were excluded from the analysis due to low RNA input or poor RNA quality. Subsequent data normalization were performed with the nSolver Analysis Software (NanoString nCounter Technologies) to correct for differences in hybridization efficiency using the respective control probes. Counts were not corrected for RNA input. Following a log^2^ transformation of the normalized data, the interquartile range (IQR) of counts for each probe across all samples in the run was calculated. Outliers in each sample, that is, positive signal for a gene fusion transcript, were determined as counts larger than 1.5*IQR, and which exceed the background threshold of 40 counts. The counts were not compared to the median of the counts across all the probes within a sample as reported by Chang et al.^7^ A comparison of both methods did not alter the results for the sample set described in this work (data not shown).

## 3 RESULTS

As shown in Figure 1 and Table 1, the NanoString assay detected gene fusions in 60/104 cases suitable for analysis. In 44/60 NanoString positive cases, a similar gene fusion had already been detected by previous alternative molecular testing. In the other 16/60 cases, no previous molecular testing had been performed. The detected fusion genes are summarized in Table S1.

**Table 1:** Overview soft tissue tumors evaluated with NanoString

Diagnosis	Total cases	NanoString fusion positive			NanoString fusion negative		
		Prior testing +	Prior testing −	No prior testing	Prior testing +	Prior testing −	No prior testing
Alveolar soft part sarcoma	3	—	—	2	—	—	1
Alveolar rhabdomyosarcoma	5	2	—	3	—	—	—
Aneurysmal bone cyst	6^a^	2	—	1	—	—	3
Angiomatoid fibrous histiocytoma	5	3	—	1	—	—	1
*BCOR*‐rearranged sarcoma	1^b^	—	—	—	1	—	—
Biphenotypic sinonasal sarcoma	3	1	—	—	—	—	2
*CIC*‐rearranged sarcoma	1	—	—	—	1	—	—
Clear‐cell sarcoma	4	3	—	—	1	—	—
Congenital/infantile fibrosarcoma	3	2	—	—	—	—	1
Desmoplastic small round cell tumor	3	3	—	—	—	—	—
Epithelioid hemangioendothelioma	5	2	—	—	3	—	—
Ewing sarcoma	8^c^	8	—	—	—	—	—
Undiff. round cell sarcoma	7	—	—	—	—	5	2
Extraskeletal myxoid chondrosarcoma	5	2	—	1	—	1	1
Inflammatory myofibroblastic tumor	7	2	—	—	2	—	3
Lipoblastoma	3	—	—	—	—	—	3
Mesenchymal chondrosarcoma	6	1	—	3	—	—	2
Myoepithelial tumor	4	—	—	—	—	1	3
Myxoid liposarcoma	7	5	—	2	—	—	—
Nodular fasciitis	5	—	—	3	—	—	2
Synovial sarcoma	8	8	—	—	—	—	—
Tenosynovial giant cell tumor	5	—	—	—	—	—	5
Total cases	104	44	0	16	8	7	29

a Two soft tissue tumors, four bone tumors.

b A bone tumor.

c Four soft tissue tumors, four bone tumors.

In 44/104 cases, no fusion was detected by the NanoString assay, whereas in 8/44 cases, a gene rearrangement or fusion had been found by prior alternative molecular testing (5 by FISH, 2 by FISH and RT‐PCR, and 1 by targeted NGS). Thus, there were no false‐positive NanoString results and eight false‐negative NanoString results. Overall, fusion gene detection by NanoString had a sensitivity of 85% and specificity of 100%.

### 3.1 Concordant and discordant (false‐negative) cases

Of the 52/104 cases, in which a gene rearrangement or fusion had been detected by prior molecular testing, NanoString was positive (concordant) in 44 cases and negative (discordant) in 8 cases. With respect to soft tissue tumor type, concordant cases included all eight Ewing sarcomas (four with *EWSR1‐FLI1*, three with *EWSR1‐ERG*, and one with *EWSR1‐FEV*), all eight synovial sarcomas with *SS18‐SSX1/2*, all seven myxoid liposarcomas with *FUS/EWSR1‐DDIT*, and all three desmoplastic small round cell tumors (DSRCT) with *EWSR1‐WT1*.

Table 2 summarizes the eight discordant cases, in which NanoString failed to detect gene fusions that were detected by other molecular methods. These discordant cases included one single BCOR‐rearranged sarcoma (with a *BCOR* (exon 15)*‐CCNB3* (exon 5) fusion gene detected by Archer) and one single CIC‐rearranged sarcoma (with *CIC* rearrangement detected by FISH). Moreover, NanoString was negative in 1/4 clear‐cell sarcomas (positive by FISH *EWSR1* break‐apart assay), 2/5 epithelioid hemangioendotheliomas (positive by FISH for *WWTR1‐CAMTA1*), and 2/4 inflammatory myofibroblastic tumors (one with *ALK* rearrangement by FISH and one with *EML4* (exon2)*‐ALK1* (exon20) by RT‐PCR).

**Table 2:** Summarize of eight discordant cases

Tumor	Case (*n*)	Alternative testing results
BCOR‐rearranged sarcoma	1	NGS found BCOR (exon15)—CCNB3 (exon 5)
CIC‐rearranged sarcoma	1	FISH found CIC‐DUX4
Clear‐cell sarcoma	1	FISH found EWS break
Epithelioid hemangioendothelioma	3	FISH found WWTR1‐CAMTA1
Inflammatory myofibroblastic tumor	2	1 case RT‐PCR found EML4 (exon2)—ALK1 (exon20), 1 case FISH found ALK positive

### 3.2 Positive NanoString results in cases without prior molecular testing

As shown in Table 1, 16 fusion‐positive cases were detected by NanoString, which had no previously molecular testing, including 2/3 alveolar soft part sarcomas, 3/5 alveolar rhabdomyosarcomas, 1/6 aneurysmal bone cysts, 1/5 angiomatoid fibrous histiocytomas, 3/6 mesenchymal chondrosarcomas, 2/7 myxoid liposarcomas, 3/5 cases of nodular fasciitis, and 1/5 extraskeletal myxoid chondrosarcomas.

### 3.3 The relative value of the NanoString assay is strongly associated with the level of diagnostic evidence in daily practice

**Figure 2:**
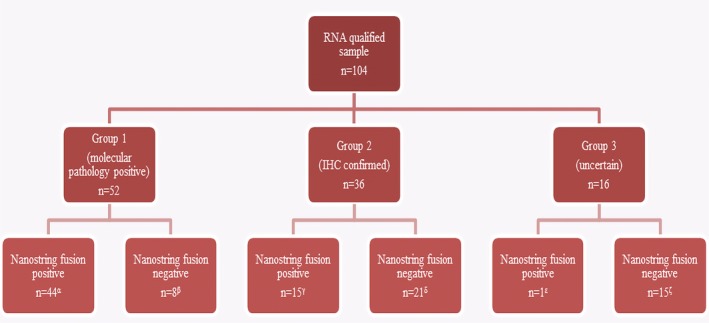
Diagnostic value of Nanostring nCounter FusionPlex in different fusion‐associated tumor types. *α*: Two alveolar rhabdomyosarcomas, two aneurysmal bone cysts, three angiomatoid fibrous histiocytomas, one biphenotypic sinonasal sarcoma, three clear cell sarcomas, two infantile fibrosarcomas, three desmoplastic small round cell tumors, two epithelioid hemangioendotheliomas, eight Ewing sarcomas, two extraskeletal myxoid chondrosarcomas, two inflammatory myofibroblastic tumors, one mesenchymal chondrosarcoma, five myxoid liposarcomas, and eight synovial sarcomas. *β*: One BCOR‐rearranged sarcoma, one CIC‐rearranged sarcoma, one clear cell sarcoma, three epithelioid hemangioendotheliomas, and two inflammatory myofibroblastic tumors. *γ*: Two alveolar soft part sarcomas, three alveolar rhabdomyosarcomas, one aneurysmal bone cyst, one angiomatoid fibrous histiocytoma, three mesenchymal chondrosarcomas, two myxoid liposarcomas, and three nodular fasciitis. *δ*: One alveolar soft part sarcoma, three aneurysmal bone cysts, one angiomatoid fibrous histiocytoma, two biphenotypic sinonasal sarcomas, one infantile fibrosarcoma, one inflammatory myofibroblastic tumor, three lipoblastomas, two mesenchymal chondrosarcomas, two nodular fasciitis, and five tenosynovial giant cell tumors. *ε*: One extraskeletal myxoid chondrosarcoma. *ζ*: Seven undifferentiated round cell sarcomas, two extraskeletal myxoid chondrosarcomas, two inflammatory myofibroblastic tumors, and four myoepithelial tumors [Color figure can be viewed at http://wileyonlinelibrary.com]

In order to determine the usefulness of NanoString testing in daily pathology practice, we divided the 104 soft tissue and bone (STB) tumors in three groups according to their level of diagnostic evidence, as shown in Figure 2. Group 1 consisted of 52 STB tumors in which the histological diagnosis was confirmed by prior alternative molecular testing. Fusion genes transcripts were detected by NanoString in 44 cases (85%). Group 2 consisted of 36 STB tumors in which the histological diagnosis was based on typical histological features, often in combination with IHC findings. Fusion gene transcripts were detected by NanoString in 15 cases (42%). Group 3 consisted of 16 STB tumors, in which the histological diagnosis was uncertain, due to overlapping or undifferentiated morphologic features and lack of specific IHC markers. In this group, a fusion gene transcript was detected by NanoString in only one case (6%), an extraskeletal myxoid chondrosarcoma with an *EWSR1‐NR4A3* fusion.

### 3.4 Estimated diagnostic coverage of NanoString in STB tumors

By combining the results of this study (Table 1) with those obtained by Chang et al.^7^ (as shown in their Table 2), it may be concluded that the NanoString nCounter assay has an excellent diagnostic coverage for five tumor types. In both studies, specific fusion genes were detected in all cases of Ewing sarcoma (n = 28), synovial sarcoma (n = 19), myxoid liposarcoma (n = 12), alveolar rhabdomyosarcoma (n = 10), and desmoplastic small round cell tumor (n = 5). Moreover, five out of six infantile fibrosarcomas were diagnosed.

Tumors with an estimated diagnostic coverage of 50% to 75% included nodular fasciitis (10/17), clear cell sarcoma (8/13), alveolar soft part sarcoma (6/8), mesenchymal chondrosarcoma (6/8), angiomatoid fibrous histiocytoma (5/7), extraskeletal myxoid chondrosarcoma (4/6), and BCOR‐rearranged sarcoma (3/4).

Tumors with a low diagnostic coverage of less than 50% included epithelioid hemangioendothelioma (4/11), myoepithelial tumors (3/9), aneurysmal bone cyst (3/9), inflammatory myofibroblastic tumor (2/9), CIC‐rearranged sarcoma (1/4), and biphenotypic sinonasal sarcoma (1/3).

Tumors in which no fusion genes were detected included (CD99 negative) undifferentiated round cell sarcomas (13), tenosynovial giant cell tumors (6), and lipoblastomas (4).

## 4 DISCUSSION

Soft tissue tumors are highly heterogeneous in histological and molecular subtypes. The identification of tumor type‐specific gene translocations has enabled a molecular classification with diagnostic and prognostic value.^8^ In this study, we demonstrate that NanoString fusion gene transcript profiling can reliably identify five molecularly defined soft tissue tumors: Ewing sarcoma, synovial sarcoma, myxoid liposarcoma, alveolar rhabdomyosarcoma, and desmoplastic small round cell tumor. Further improvement of the assay can likely extend its diagnostic value to other sarcoma subtypes.

The diagnostic coverage of the current design of the NanoString panel for the other relatively rare tumor types included in this study is limited. The most likely reason for this is the lack of probes for known and unknown gene fusion events. Furthermore, lack of performance was demonstrated for a few probes in the current design. The probe for *EML4* (exon2)*‐ALK* (exon 20) did not identify this gene fusion event in two inflammatory myofibroblastic tumors that were previously determined by RT‐PCR and FISH. However, analysis of these samples with the commercially available lung carcinoma fusion gene panel did demonstrate this transcript in these tumors (data not shown).

For other previously identified translocations that could not be confirmed with the current NanoString panel, it is unknown whether this is due to a lack of performance of the fusion gene probes or a lack of probes for other known and unknown fusion. For example, this study included one CIC‐rearranged sarcoma in which a *CIC* rearrangement was demonstrated by FISH previously. However, a *CIC*‐*DUX4* fusion gene transcript could not be detected. It is estimated that *CIC‐DUX4* fusions can be observed in approximately 60% of CIC‐rearranged sarcomas, with lower incidence of others fusion partner such as *FOXO4* and *NUTM1*.^9^, ^10^ Chang et al.^7^ demonstrated that the current NanoString panel identified one *CIC*‐*DUX4* fusion transcript in four CIC‐rearranged sarcomas, indicating that at least one of the probes is working. Therefore, and in contrast to well‐studied soft tissue tumors such as Ewing sarcoma and synovial sarcoma, the current panel design has a high false negative rate for rare tumors in which the gene‐fusion partners and exact location of the break are poorly characterized. The combined analysis of the current and previously published study^7^ indicates, with the exception Ewing sarcoma, synovial sarcoma, myxoid liposarcoma, alveolar rhabdomyosarcoma, and desmoplastic small round cell tumors, a moderate to high risk of a false negative result (25% and higher, depending on tumor type).

The current panel appears not suitable for the molecular analysis of undifferentiated round cell carcinoma, lipoblastoma, and tenosynovial giant‐cell tumors. Despite the inclusion of probes for fusion genes frequently detected in these tumors, none were positive in the NanoString analysis.

In addition to the tumor type‐specific performance of this NanoString test, the percentage of tumor cells in a sample as well as RNA quality can contribute to a false‐negative test result. Although the minimal percentage of tumor cells in a sample that is required for a confident detection of a gene fusion transcript was not determined, only samples with >50% tumor cellularity were included. Furthermore, only samples from which at least 15 ng RNA/μL could be extracted were analyzed with NanoString using 100 ng RNA input. Some samples were analyzed with 300 ng RNA input, but that did not result in a higher diagnostic yield (data not shown). Despite a high RNA yield from one desmoplastic small round cell tumor and one undifferentiated round cell sarcoma, counts for the reference genes were insufficient for analyses. Re‐examination of both tissues revealed extensive necrosis that was presumably causal to poor RNA quality. Therefore, irrespective of sufficient tumor cellularity and RNA yield, a NanoString analysis can fail due to poor RNA quality.

The cost effectiveness and short turn‐around time of a NanoString analysis is a strong argument for the replacement of FISH and RT‐PCR as the initial screening test for sarcomas.^5^ Turnaround time for FISH and a NanoString assay in a diagnostic setting is comparable, yet a NanoString assay is less labor intensive. In agreement with Chang et al.,^7^ the cost per sample of a FISH analysis (one target‐one sample) is comparable to one multiplex NanoString analysis when analyzing 12 samples simultaneously. NanoString thus significantly reduces the cost per sample while maintaining a short turnaround time. However, when no fusion event is identified, additional molecular profiling based on, for example, multiplex PCR (AMP)‐based NGS may be necessary. This will be required for those tumors for which the current NanoString panel has a low diagnostic yield. For these tumor types, analysis Archer RNA‐seq NGS is likely more effective,^11^ but is associated with higher costs and longer turnaround times that are comparable to NGS sequencing of large targeted panels.

In conclusion, the NanoString nCounter FusionPlex assay is a screening tool with high sensitivity and specificity^5^, ^6^, ^7^, ^12^, ^13^ for the detection of sarcoma‐defining fusion gene transcripts in Ewing sarcoma, synovial sarcoma, myxoid liposarcoma, alveolar rhabdomyosarcoma, and desmoplastic small round cell tumors. Its diagnostic yield for rare soft tissue tumors is limited and might require additional or alternative testing.

## Supporting information


**Table S1.** Validated fusion genes using NanoString nCounter FusionPlex assay

## Data Availability

The data that support the findings of this study are available on request from the corresponding author. The data are not publicly available due to privacy or ethical restrictions.
