# Supplementary material for: Diagnostic yield of NanoString nCounter FusionPlex profiling in soft tissue tumors
Source: Genes Chromosomes Cancer. 2020 Jan 31;59(5):318–24. doi: 10.1002/gcc.22834 (PMC7079105; doi:10.1002/gcc.22834)
Supplement: Supplementary file 1 — Table S1. Validated fusion genes using NanoString nCounter FusionPlex assay [file GCC-59-318-s001.docx]

Supplemental Table 1: Validated fusion genes using NanoString nCounter FusionPlex assay

| Diagnosis | Fusion genes |
| --- | --- |
| Alveolar soft part sarcoma | ASPSCR1(Exon2)-TFE3(Exon3),  ASPSCR1(Exon2)-TFE3(Exon4) |
| Alveolar rhabdomyosarcoma | PAX3(Exon7)-FOXO1(Exon2),  PAX7(Exon7)-FOXO1(Exon2) |
| Aneurysmal bone cyst | COL1A1(Exon1)-USP6(Exon2) |
| Angiomatoid fibrous histiocytoma | EWSR1(Exon7)-AFT1(Exon5),  EWSR1(Exon7)-CREB1(Exon7) |
| Biphenotypic sinonasal sarcoma | PAX3(Exon7)-FOXO1(Exon2) |
| Clear-cell sarcoma | EWSR1(Exon8)-AFT1(Exon4) |
| Congenital fibrosarcoma | ETV6(Exon5)-NTRK3(Exon15) |
| Desmoplastic small round cell tumor | EWSR1(Exon7)-WT1(Exon8) |
| Epithelioid hemangioendothelioma | RANBP2(Exon18)-ALK(Exon20),  WWTR1(Exon3)-CAMTA1(Exon9) |
| Ewing sarcoma | **Probe detects 2 EWS-ERG variants*:**  EWSR1(Exon7)-ERG(Exon11),  EWSR1(Exon7)-ERG(Exon7),  EWSR1(Exon7)-FLI1(Exon5),  EWSR1(Exon7)-ERG(Exon6),  FUS(Exon10)-FEV(Exon2),  EWSR1(Exon7)-FLI1(Exon6) |
| Extraskeletal myxoid chondrosarcoma | **Probe detects 2 EWS-** **NR4A3 variants*:**  EWSR1(Exon12)-NR4A3(Exon3),  EWSR1(Exon13)-NR4A3(Exon3) |
| Inflammatory myofibroblastic tumor | EML4(Exon2)-ALK(Exon20),  SEC31A(Exon22)-ALK(Exon21) |
| Mesenchymal chondrosarcoma | HEY1(Exon4)-NCOA2(Exon13) |
| Myxoid liposarcoma | EWSR1(Exon10)-DDIT3(Exon2),  EWSR1(Exon13)-DDIT3(Exon2),  EWSR1(Exon7)-DDIT3(Exon2),  FUS(Exon5)-DDIT3(Exon2) |
| Nodular fasciitis | COL1A1(Exon1)-USP6(Exon2),  MYH9(Exon1)-USP6(Exon1partial),  MYH9(Exon1)-USP6(Exon2) |
| Synovial sarcoma | **Probe detects 3 SYT-SSX variants*:**  SS18(Exon10)-SSX1(Exon5),  SS18(Exon10)-SSX1/2(Exon6),  SS18(Exon10)-SSX4(Exon6) |

*: Due to alternative splicing, some tumors presented different fusion variants in the same tumor.
